# Increased abundance of bacteria of the family Muribaculaceae achieved by fecal microbiome transplantation correlates with the inhibition of kidney calcium oxalate stone deposition in experimental rats

**DOI:** 10.3389/fcimb.2023.1145196

**Published:** 2023-05-29

**Authors:** Yan Wang, JinBo Sun, Sen Xie, Yu Zhou, Tao Wang, ZhenYu Liu, ChaoSheng Li, Lei Gao, TieJun Pan

**Affiliations:** ^1^ Department of Urology, General Hospital of Central Theater Command of Chinese People’s Liberation Army, Wuhan, Hubei, China; ^2^ The First School of Clinical Medicine, Southern Medical University, Guangzhou, Guangdong, China

**Keywords:** nephrolithiasis, fecal microbiome transplantation, Muribaculaceae, oxalate-degrading bacteria, renoprotective function, microbial network

## Abstract

**Background:**

The incidence of nephrolithiasis is increasing rapidly worldwide. Calcium oxalate is the most common constituent, contributing to approximately 80% of all kidney stones. The gut microbiome, through its oxalate-degrading ability, may play a role in decreasing morbidity due to urinary calculus. Fecal microbiome transplantation (FMT) has been reported to be effective in restoring the gastrointestinal microbial community in different conditions. The transplantation of whole communities that have oxalate-degrading function may be a more effective strategy than the transplantation of isolated strains.

**Methods:**

FMT was carried out in male guinea pigs and male Sprague–Dawley laboratory rats (SDRs). Fresh feces were collected from guinea pigs housed in metabolic cages. SDRs were divided into four groups: two groups received standard rat chow (SC) (groups SC and SC + FMT), and two groups were fed a 5% potassium oxalate diet (OD) (groups OD + phosphate-buffered saline (PBS) and OD + FMT). On day 14, groups OD + PBS, OD + FMT, and SC + FMT received either PBS or guinea pig feces by esophageal gavage. The composition of the microbiota of guinea pigs and SDRs was analyzed using a 16S rRNA gene sequencing approach. Biochemical analysis of urine samples from SDRs revealed the presence of calcium oxalate (CaOx) crystals, which were presumed to originate from kidney stones. Renal function was examined using real-time PCR analysis and immunohistochemical staining for renin, angiotensin-converting enzyme, and osteopontin (OPN) expression.

**Results:**

FMT resulted in a gut microbiota that was a mixture of guinea pig and SDR bacteria. A microbial network involving Muribaculaceae, *Lactobacillus*, and *Bifidobacterium* was activated by FMT in group OD + FMT. As a result, urinary oxalate, calcium, uric acid, creatinine and urea in urine samples were reduced significantly. Similarly, significant reduction of uric acid and blood urea nitrogen to creatinine ratio in serum samples was observed (*p* < 0.05). Microscopic observations revealed a high CaOx crystal score (4+) in the kidneys of rats in group OD + PBS, whereas a lower score (2+) was observed in the rats in group OD + FMT. Up-regulation of OPN and down-regulation of renin were also associated with FMT.

**Conclusion:**

A microbial network involving Muribaculaceae and other oxalate-degrading bacteria achieved by FMT was capable of reducing urinary oxalate excretion and CaOx crystal deposition in the kidney through increasing intestinal oxalate degradation. FMT may exert a renoprotective function in oxalate-related kidney stones.

## Introduction

As one of the most common urinary diseases, kidney stone disease places a tremendous burden on public health, with a recurrence rate of 50% within 5 years. Approximately 80% of all kidney stones are composed of calcium oxalate (CaOx). Oxalate is endogenously produced in the liver from ingested or metabolically generated precursors and is also absorbed in the intestine from an oxalate-containing diet (Holmes et al., 2001; Jaeger and Robertson, 2004; Knight et al., 2006; Taylor and Curhan, 2008). Secondary hyperoxaluria, which is a result of intestinal hyperabsorption of oxalate or a high intake of oxalate, is considered a crucial risk factor in CaOx stone formation (Curhan et al., 2001). Mammals that lack certain enzymes cannot degrade oxalate. The current clinical treatment for kidney stones is mainly surgical, which is effective in the short term; however, surgery inevitably causes damage to the kidneys.

The gut microbiota is closely associated with health maintenance and the pathogenesis of various diseases. Differences in gut microbiota between kidney stone patients and healthy individuals were detected using a 16S rRNA gene sequencing approach. Stern et al. analyzed the gut microbiota of 23 kidney stone patients and six control subjects without kidney stones. They found that *Bacteroides* was 3.4 times more abundant in the kidney stone group than in the control group, and *Prevotella* was 2.8 times more abundant in the control group than in the kidney stone group (Stern et al., 2016). A case–control study involving 52 kidney stone formers and 48 control subjects was conducted to compare intestinal microbiota composition and functionality. The results showed that kidney stone formers exhibited lower intestinal microbial diversity than control subjects. In addition, bacterial representation of genes involved in oxalate degradation was significantly lower in kidney stone formers than in control subjects (Ticinesi et al., 2018). Similarly, Tang et al. found that the microbial diversity, taxonomic composition, and functional potential of the intestinal microbiota were significantly different in patients with kidney stones and healthy control subjects (Tang et al., 2018). In summary, the gut microbiota, especially oxalate-degrading bacteria, has potential value in the prevention and treatment of kidney stone disease.

Oxalate-degrading bacteria can be categorized into two groups. Obligate oxalate consumers, such as *Oxalobacter formigenes*, use oxalate as their sole energy source (Allison et al., 1985). Facultative oxalate consumers, in contrast, which include species of the genera *Lactobacillus* (Turroni et al., 2010), *Bifidobacterium* (Mogna et al., 2014), and *Enterococcus*, can also use other energy sources. *O. formigenes* possesses a specialized set of enzymes capable of degrading oxalate and preventing its absorption into the circulation (Baetz and Allison, 1989; Baetz and Allison, 1990). In addition, *O. formigenes* can stimulate oxalate secretion from the circulation back into the gut (Hatch et al., 2006). As a result, urinary oxalate excretion, which acts as an important influencing factor in the formation of calcium oxalate stones, is reduced. Many studies have focused on the use of oxalate-degrading bacteria as probiotic preparations to treat kidney stones. Such probiotic formulations can comprise *O. formigenes* alone or in different combinations with *Lactobacillus*, *Bifidobacterium*, and *Enterococcus* species and other facultative oxalate degraders. However, several studies (Goldfarb et al., 2007; Hoppe et al., 2011; Siener et al., 2013) have shown that probiotic formulations are ineffective in reducing urinary oxalate excretion, or have only a temporary effect (Hoppe et al., 2017; Milliner et al., 2018). Oxalate metabolism by the gut microbiota may not be limited to oxalate-degrading bacteria, and a network of bacteria that can metabolize oxalate may exist in the gut. The transplantation of whole communities adapted to oxalate degradation is a more effective way of transferring oxalate-degrading function than the transplantation of isolated strains. Miller et al. transplanted a whole-community microbiota from a wild mammalian herbivore, *Neotoma albigula*, to Sprague–Dawley laboratory rats (SDRs). The microbial transplants resulted in a significant increase in oxalate degradation (Miller et al., 2016). In addition, Miller et al. examined the reduction in oxalate excretion in a rat model following oral administration of fecal microbes from *Neotoma albigula* or formulations of mixed oxalate-degrading isolates, and a better effect was seen with fecal microbial transplants (Miller et al., 2017a).

Allison et al. found that rates of oxalate degradation by microbes in the gastrointestinal contents of herbivores such as rabbits, guinea pigs, swine, and horses increased after the addition of oxalate to diets. The concentration of oxalate-degrading microbes, which are normally present in the large bowel of rabbits, guinea pigs, horses, and swine, was found to increase in response to the increased availability of oxalate (Allison and Cook, 1981). Argenzio et al. also found that microorganisms present in the large intestine of guinea pigs were capable of degrading oxalate (Argenzio et al., 1988). Adaptation of animals to a high-oxalate diet provided protection against an acute oxalate load and reduced the fractional absorption of oxalate. However, the level of oxalate degradation in SDRs decreased with continued exposure to oxalate, which is indicative of maladaptation of their native gut microbiota to oxalate consumption (Sidhu et al., 2001). Thus, these two species, guinea pigs and SDRs, are excellent models to examine the effect of fecal transplants on oxalate degradation.

The aim of the present study was to examine the role of whole-community microbial transplants in the transfer of oxalate degradation capacity between species. First, the whole microbiota was transferred from guinea pigs to another rodent, SDRs. Second, the effectiveness of transplantation and the oxalate-degrading ability of recipients were determined. Furthermore, we preliminarily explored the renoprotective role of the gut microbiota and found that whole-community microbial transplants repaired oxalate-induced kidney impairment to some extent.

## Methods

### Animals and study design

Male SDRs (140–160 g, 5 weeks) and male guinea pigs (300–350 g, 8 weeks) were purchased and kept in the experimental animal center of Wuhan University. The experimental procedure was performed in accordance with the animal experimentation guidelines of Wuhan University. The protocols for animal experiments were approved by the Animal Ethics Committee of Wuhan University (no. WP20220064). All animals were kept at 27 ± 2°C with a 12-h light/dark cycle. After being fed a 1.5% oxalate diet for 2 weeks, guinea pigs were put into metabolic cages, and fresh feces were collected within 6 hours (Cammarota et al., 2017). This approach helps to minimize microbiota exposure time to aerobic conditions. Fecal microbiome transplantation (FMT) was conducted in accordance with protocols from previous studies (Miller et al., 2016; Stern et al., 2019). Feces from guinea pigs were mixed and ground with a sterilized pestle and mortar. Powdered feces were dissolved in saline to make a suspension containing 5% feces. The SDRs were randomly divided into four groups (*n* = six per group). Two groups received standard chow (SC) (groups SC and SC + FMT), while two experimental groups were fed a 5% potassium oxalate diet (OD) to induce hyperoxaluria (groups OD + PBS and OD + FMT). The chow with oxalate content was purchased from Beijing Keao Xieli Feed Co., Ltd, and was customized by supplementing powdered potassium oxalate. SDRs and guinea pigs were fed the oxalate-containing chow in the form of cylindrical pellets. From day 14, rats in group OD + PBS were administered, by esophageal gavage, 2 mL of PBS daily, while rats in groups OD + FMT and SC + FMT were administered 2 mL of the 5% feces suspension daily. The SDRs were sacrificed on day 31. Serum samples were obtained for biochemical analysis, and kidney tissues were harvested and processed for the localization of crystals and various other morphological analyses.

### Feces collection and microbiome analysis

Fresh feces were collected from guinea pigs that had been housed in metabolic cages and fed a 1.5% oxalate diet for 14 days. On day 30, all SDRs were placed in metabolic cages so that fresh feces could be collected. Feces were frozen at –80°C until DNA extraction. Microbial community genomic DNA was extracted from the collected fecal samples using a Magnetic Soil And Stool DNA Kit (Tiangen Biotech Co., Ltd, Beijing) in accordance with the manufacturer’s instructions. The DNA extract was identified on 1% agarose gel, and DNA concentration and purity were determined with NanoDrop 2000 UV-Vis spectrophotometer (Thermo Fisher Scientific, Wilmington, DE, USA). The hypervariable region (V4) of the bacterial 16S rRNA gene was amplified with primers 341F and 806R. Operational taxonomic units (OTUs) with a 97% similarity cutoff were clustered using UPARSE version 7.1, and chimeric sequences were identified and removed. QIIME 2™ was used to analyze species composition, alpha diversity, and beta diversity, to determine linear discriminant analysis effect size, and for the creation of Venn diagrams and Manhattan plots.

### Urine collection and analysis

On day 30, the SDRs were placed in metabolic cages to enable the collection of a 24-h urine sample. A commercial semiautomatic photometer was used in accordance with the manufacturer’s protocol to determine urinary oxalate, uric acid, creatinine, urea, and calcium. One-hour urine samples were collected on day 28 and examined by polarized light microscopy to analyze the presence of CaOx crystalluria.

### Serum parameters analysis

Serum parameters, such as uric acid, creatinine, urea, and calcium, were measured using the appropriate kits and in accordance with the manufacturer’s instructions.

### RNA isolation and quantitative real-time PCR

Total RNA was isolated from kidney tissue (50–100 mg) using a commercial kit in accordance with the manufacturer’s recommendations (TransGen Biotech Co., Ltd, Beijing, China). Extracted RNA samples were tested for quality and quantity. Subsequently, reverse transcription reactions were performed before further quantitative real-time PCR (qRT-PCR) analysis. The levels of mRNA for glyceraldehyde-3-phosphate dehydrogenase (GAPDH), renin, angiotensin-converting enzyme (ACE), and osteopontin (OPN) in the kidney were then quantified by qRT-PCR. Primers of GAPDH, renin, ACE, and OPN can be seen in **Table S1**.

### Analysis of histopathology and CaOx crystals in the kidney

The rats were sacrificed at the end of the experiments and kidney tissues were obtained. After treatment with 10% formalin, kidney specimens were trimmed to an optimal size and subsequently immersed in paraffin. Three-μm-thick paraffin sections were cut and subjected to hematoxylin and eosin staining to evaluate the histological and morphological changes. The stained sections were then observed under a light microscope to assess renal morphology. The presence of CaOx crystals was scored on a scale of 0–5+. Pizzolato staining was conducted to assess CaOx crystals formed in renal tissues, and a polarized light microscope was used to visualize the CaOx crystals. Pathological analysis was performed under the guidance of an experienced pathologist.

### Immunohistochemistry

Representative 3-μm kidney tissue slices were obtained and subjected to dewax and rehydration in xylene and graded ethanol solutions. Heat-based antigen retrieval was performed in citrate buffer by microwave. Subsequently, tissue slices were incubated with 3% hydrogen peroxide for the inactivation of endogenous peroxidases. After incubation with a blocking solution to block non-specific binding, slices were treated with anti-ACE (SA1022, Boster Biological Technology, Pleasanton, CA, USA, dilution 1:800), anti-OPN (YT3467, ImmunoWay Biotechnology Company, Plan, TX, USA, dilution 1:300), and anti-renin (YT4047, ImmunoWay Biotechnology Company, dilution 1:100). Subsequently, the slices were exposed to horseradish peroxidase-linked secondary antibody (RS0002, ImmunoWay Biotechnology Company, dilution 1:200). Finally, diaminobenzidine staining and hematoxylin counterstaining were performed to visualize antigen binding. An Olympus BX51 microscope was used for image capturing.

### Statistical analysis

Data were expressed as mean ± SD. The statistical significance of differences between groups was analyzed with a one-way ANOVA using SPSS version 23 software. Results were considered significant if the *p*-value was < 0.05.

## Results

### The gut microbiome varies between guinea pigs and rats

**Figure 1:**
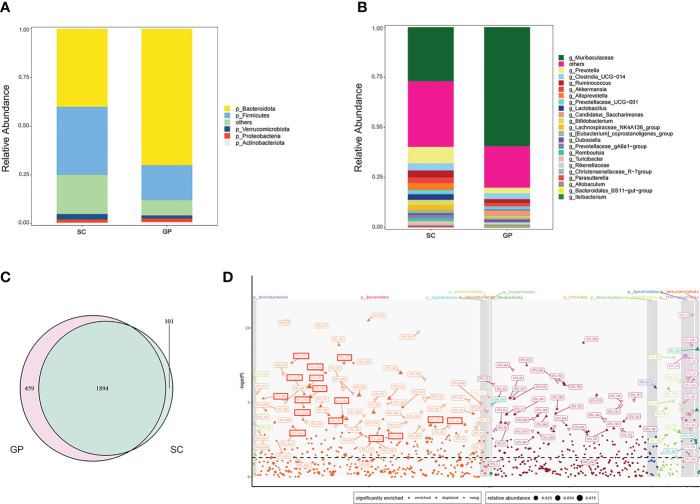
Characterization of the gut microbiota between groups of guinea pigs and Sprague–Dawley laboratory rats (SDRs). **(A)** Phylum level of the microbiota in group SC rats and guinea pigs. **(B)** Genus level of the microbiota in group SC rats and guinea pigs. **(C)** Venn diagram showing shared and exclusive operational taxonomic units (OTUs) for group SC rats and guinea pigs. **(D)** Manhattan plot demonstrating changes in OTUs in guinea pigs that received an oxalate mixed diet for 2 weeks. The shape of the dots in the graph indicates the type of change in the relative abundance of OTUs, i.e., whether it is enriched (positive solid triangles) or depleted (inverted hollow triangles), or there is no significant difference in change (solid dots). The OTUs boxed in red represent Muribaculaceae. Straight lines refer to OTUs represented by the different shapes of dots.

To explore the gut microbiota diversity between guinea pigs and SDRs, we tried to establish a detailed and comparative gut microbial profile. By analyzing fecal samples using 16S rRNA bacteria gene sequencing, we obtained 5,263,290 paired-end, high-quality 16S V4 region sequence reads with a median read length of 231 and an average of 146,202 ± 10,496 reads per sample. A total of 47,257 OTUs were defined by 97% similarity across all fecal samples. Based on rarefaction analysis, 121,990 sequence reads provided a good estimate of true diversity. Our results indicated that the species composition of microbiota differed significantly between guinea pigs and SDRs. A Venn diagram revealed the presence of unique OTUs in guinea pigs and SDRs in addition to the 1,894 OTUs shared by two groups (**Figure 1C**). At the phylum level, a higher relative abundance of Bacteroidetes was detected in guinea pigs than in SDRs (**Figure 1A**). More importantly, we found that the relative abundance of Muribaculaceae was significantly increased (mean relative abundance 27% vs. 52%, *p* < 0.05) at the genus level in guinea pigs (**Figure 1B**). Species of the family Muribaculaceae, belonging to the phylum Bacteroides, have been reported to be able to degrade oxalate (Ormerod et al., 2016; Lagkouvardos et al., 2019). Therefore, a Manhattan plot was created to visualize variation in the gut microbiota of guinea pigs that received an oxalate-containing diet. We found that an oxalate-containing diet resulted in a significant increase in the relative abundance of 69 OTUs, including Muribaculaceae (**Figure 1D**). The results indicated that a Muribaculaceae-containing microbial network in the intestine of guinea pigs may be responsible for oxalate metabolism.

### Fecal microbiome transplantation affects the gut microbiota

**Figure 2:**
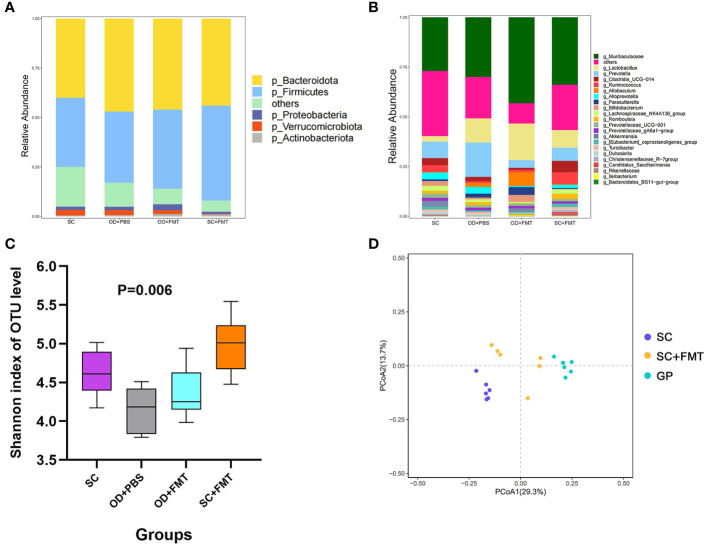
Effects of fecal microbiome transplantation. **(A)** Phylum level of the microbiota among the four groups of rats. **(B)** Genus level of the microbiota among the four groups of rats. **(C)** Shannon diversity index of the four groups of rats. **(D)** Principal coordinate analysis plot based on an unweighted unique fraction metric analysis depicts the effect of fecal transplants on the microbial community of Sprague–Dawley laboratory rats that received standard chow.

The species composition of the gut microbiota was different among the four groups of rats (**Figures 2A, B**). The Shannon diversity index was used to estimate microbial diversity in samples. An increase in the Shannon diversity index in group SC + FMT compared with group SC revealed that FMT could enhance the diversity of intestinal microbial species in rats. A similar result was observed in groups OD + PBS and OD + FMT (**Figure 2C**). As shown in a principal coordinate analysis plot based on an unweighted unique fraction metric analysis, the gut microbiota of group SC + FMT drifted away from group SC and was similar to group Guinea Pig (GP) after the fecal transplant (**Figure 2D**). In group SC + FMT, FMT resulted in a gut microbiota that was a mix of guinea pig and SDR bacteria. These changes in the gut microbiota illustrate that FMT was effective in transferring oxalate-degrading ability in experimental rats that did not receive an oxalate mixed diet.

**Figure 3:**
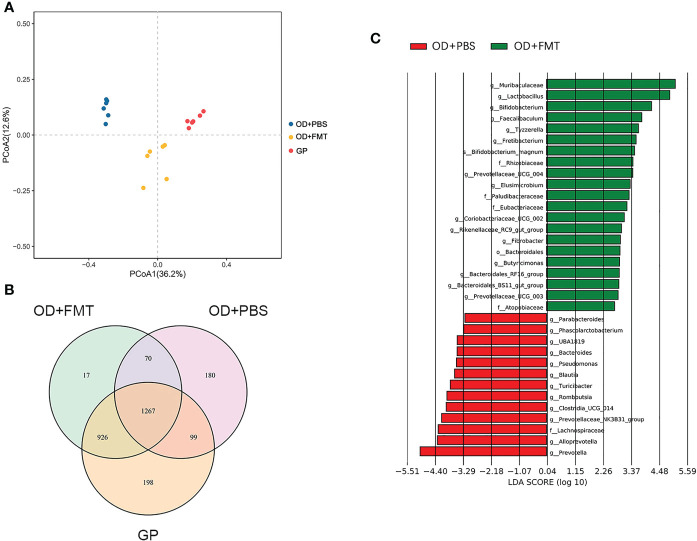
Microbiota altered by fecal microbiome transplantation. **(A)** Principal coordinate analysis plot based on an unweighted unique fraction metric analysis depicts the effect of whole microbial community transplantation on gut microbiota in Sprague–Dawley laboratory rats (SDRs) that were fed an oxalate mixed diet. **(B)** Venn diagram showing the operational taxonomic units of fecal transplant and no-transplant groups of SDRs and guinea pigs. **(C)** Linear discriminant analysis diagram showing the microbiota that are enriched in the intestine of rats in groups OD + FMT and OD + PBS.

We also examined rats fed an oxalate mixed diet and analyzed the changes in gut microbiota brought about by FMT. A total of 926 OTUs were unique to groups OD + FMT and GP, while 1,267 OTUs were shared by all groups (**Figure 3B**). FMT increased the number of OTUs shared by the microbiota of SDRs and guinea pigs. This finding is consistent with the gut microbiota drift from initial SDR microbiota to guinea pig microbiota in SDRs that received microbial transplants (**Figure 3A**). The differentially abundant taxa between groups SC and SC + FMT were identified by linear discriminant analysis (LefSe), and we found that 15 genera, including *Lactobacillus*, *Bifidobacterium*, and genera of the family Muribaculaceae, were enriched in group SC + FMT (**Figure 3C**). Interestingly, Muribaculaceae, *Lactobacillus*, and *Bifidobacterium* species are all oxalate-degrading bacteria. Thus, our findings show that FMT can facilitate the establishment of a microbial network involving Muribaculaceae and other oxalate-degrading bacteria in the intestine of SDRs that receive an oxalate mixed diet.

### Fecal microbiome transplantation reduces urinary oxalate excretion

**Figure 4:**
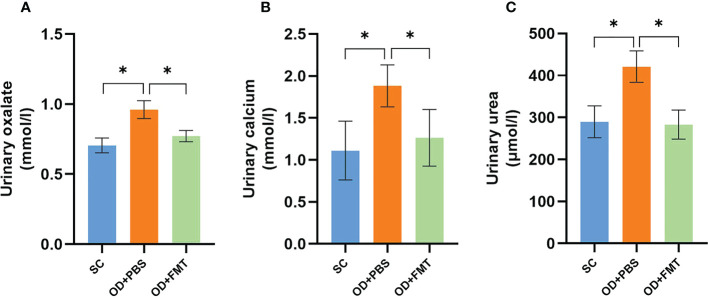
Urinary oxalate, calcium, and urea excretions in three groups of rats. **(A)** Urinary oxalate excretions in groups SC, OD + PBS, and OD + FMT. **(B)** Urinary calcium excretions in groups SC, OD + PBS, and OD + FMT. **(C)** Urinary urea excretions in groups SC, OD + PBS, and OD + FMT. One-way ANOVA was applied. *p* > 0.05, *p* < 0.05 (*).

We compared the urinary oxalate excretion in three groups of SDRs to explore the effect of FMT on oxalate metabolism. Excretion of urinary oxalate in group OD + PBS showed a significant increase in comparison with group SC. When compared with group OD + PBS, the urinary oxalate excretion of rats in group OD + FMT showed a significant reduction (*p* < 0.05; **Figure 4A**). The results indicated that FMT effectively reduced the high excretion of urinary oxalate caused by a 5% potassium oxalate diet. This reduction may be associated with enhanced degradation of oxalate by microbiota in the intestine. Similarly, a significant reduction in urinary calcium was observed in group OD + FMT compared to group OD + PBS (**Figure 4B**). In addition, we found that the urea level was higher in group OD + PBS than in group SC (*p* < 0.05). However, a significantly decreased level of urea was observed in group OD + FMT relative to group OD + PBS (**Figure 4C**).

### Prevention of crystalluria and the formation of renal CaOx crystals in rats treated with fecal microbiome transplantation

**Figure 5:**
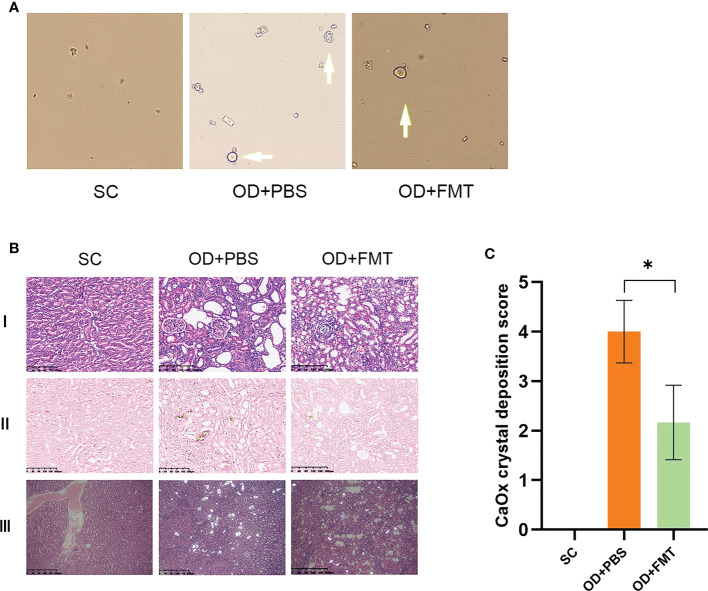
Microscopic examination of calcium oxalate (CaOx) crystals in experimental rat urine and kidneys at ×20 magnification. **(A)** Arrow indicates the CaOx crystals in the urine sample of the rat group. **(B)** CaOx crystal deposition score of the three groups of rats. *Mean value is significantly different at *p* < 0.05. **(C)** I, II, and III represent the hematoxylin and eosin-stained section, the Pizzolato methods-stained section for CaOx crystals, and the polarized microscopy examination of CaOx crystals, respectively. SC, OD + PBS, and OD + FMT indicate the different groups of rats.

After FMT, the presence of CaOx crystals in urine was examined in all rats. Rats in group SC did not show any CaOx crystals. Rats in group OD + FMT showed fewer CaOx crystals than rats in group OD + PBS (**Figure 5A**). The renal CaOx crystals were subjected to Pizzolato staining and observed under polarized microscopy. Our results revealed no deposition of CaOx crystals in group SC, whereas a high CaOx crystal score (4+) was detected in group OD + PBS (**Figure 5C**). Compared with rats in group OD + PBS, rats in group OD + FMT showed a significantly lower score (2+). We performed hematoxylin and eosin staining of kidney sections to evaluate renal morphology. The results showed that an oxalate diet induced tubular dilatation, tubular atrophy, and widening of the interstitial space in rats in group OD + PBS. However, FMT significantly attenuated tubular dilatation and tubular atrophy, suggesting an improved renal morphology in rats in group OD + FMT (**Figure 5B**).

### Fecal microbiome transplantation improves the primary health of hyperoxaluric rats

Control rats (group SC) that received SC and experimental rats (groups OD + PBS and OD + FMT) that received an oxalate mixed food gained weight and stayed healthy. However, rats in groups OD + PBS and OD + FMT gained less weight than the controls (**Table 1**). Rats in group OD + FMT gained more weight than rats in group OD + PBS, even though both groups received the 5% oxalate diet (*p* < 0.05). Several indicators in blood and urine were calculated to predict renal function. The urinary excretion of creatinine was significantly higher in groups OD + PBS and OD + FMT than in group SC (*p* < 0.05). However, the urinary creatinine level was significantly lower in group OD + FMT than in group OD + PBS (**Table 1**). The excretion of uric acid was significantly higher in group OD + PBS than in groups SC and OD + FMT (**Table 1**). The blood urea nitrogen to creatinine (BUN/creatinine) ratio was 49.33 ± 1.97 in group OD + PBS, 44.21 ± 2.21 in group SC, and 41.33 ± 2.50 in group OD + FMT, which was significantly lower than that in group OD + PBS (*p* < 0.05; **Table 2**). Moreover, the urinary uric acid level was markedly higher in group OD + PBS than in group SC (*p* < 0.05). However, the urinary uric acid concentration was significantly lower in group OD + FMT than in group OD + PBS (*p* < 0.05; **Table 2**). The significantly lower excretion of urinary urea, urinary uric acid, urinary creatinine, serum BUN/Creatinine ratio and serum uric acid in group OD + FMT revealed an improved renal function, suggesting that oxalate-mediated renal damage was attenuated by FMT.

**Table 1:** Urinary biochemistry profile.

Day	Group SC (*n* = 6)	Group OD + PBS (*n* = 6)	Group OD + FMT (*n* = 6)
Body weight (g)			
0	146.00 ± 6.16	147.17 ± 1.47	147.33 ± 5.24
7	237.55 ± 9.24	219.02 ± 4.88^#^	220.53 ± 6.84
14	335.20 ± 23.29	312.52 ± 4.38	314.37 ± 12.46
21	356.17 ± 23.74	341.42 ± 3.95	349.33 ± 9.89
28	417.50 ± 19.11	375.50 ± 9.54^#^	395.17 ± 14.34^&^
Uric acid (mmol/l)			
28	0.21 ± 0.05	0.45 ± 0.08^#^	0.31 ± 0.05^&^
Creatinine (mmol/l)			
28	2.17 ± 0.56	3.94 ± 0.76^#^	3.14 ± 0.40^&^

Data are expressed as mean ± SD. ^#^ and ^&^ indicate that the mean value is significantly different at p < 0.05 against group SC and group OD + PBS, respectively. Six rats in each group.

**Table 2:** Serum profile.

Parameter	Group SC (*n* = 6)	Group OD + PBS (*n* = 6)	Group OD + FMT (*n* = 6)
**Uric acid (μmol/L)**	55.54 ± 13.45	77.21 ± 10.33^#^	63.06 ± 11.17
**Calcium (mmol/L)**	0.91 ± 0.09	1.14 ± 0.12	1.03 ± 0.12
**BUN/creatinine ratio**	44.21 ± 2.21	49.33 ± 1.97^#^	41.33 ± 2.50^&^

Data are expressed as mean ± SD. Symbols indicate that the mean value is significantly different (p < 0.05) from ^#^group SC or ^&^group OD + PBS. Six rats in each group.

### Fecal microbiome transplantation alleviates kidney stone-induced damage in hyperoxaluric rats

**Figure 6:**
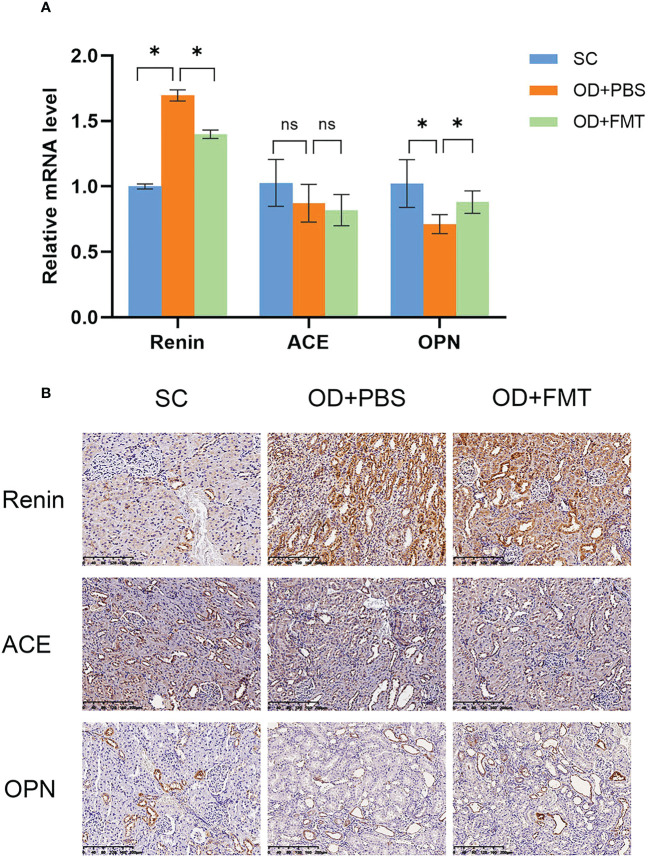
Expression of renin, angiotensin-converting enzyme, and osteopontin in the kidney. **(A)** Gene expression analysis using real-time PCR. The fold change values represent the means ± SEM (*n* = 6) in the bar diagram. SC, OD + PBS, and OD + FMT are the different rat groups. **(B)** Representative diaminobenzidine immunohistochemistry-stained sections of the kidney at ×20 magnification. *p < 0.05; ns, not significant.

To further investigate the molecular alterations in the kidney, we examined the levels of several renoprotection-associated markers, including renin, ACE, and OPN. A remarkable increase in renin mRNA expression was observed in rats in group OD + PBS compared with rats in group SC (**Figure 6A**), indicating that the rat kidneys were subjected to oxalate mixed diet-induced stress. However, the FMT-treated rats in group OD + FMT showed a significantly decreased mRNA level of renin compared to rats in group OD + PBS. The decreased renin mRNA expression indicated attenuated oxalate stress that may have resulted from enhanced oxalate degradation mediated by FMT. The mRNA levels of ACE and OPN were down-regulated in both group OD + PBS and group OD + FMT compared with group SC (**Figure 6A**). After treatment with FMT, the down-regulation of OPN mRNA expression was greatly restored in group OD + FMT in comparison with group OD + PBS. It has been reported that OPN can inhibit the formation of CaOx crystals in the kidney (Wang et al., 2008; Chien et al., 2009; Langdon et al., 2009). Increased mRNA expression of OPN illustrated that the ability to prevent crystallization of CaOx calculus was restored to some extent. To further validate the ameliorative role of FMT in kidney stone-induced damage in hyperoxaluric rats, we performed immunohistochemical staining to evaluate the protein levels of renin, ACE, and OPN (**Figure 6B**). Consistent with the mRNA expressions, the staining intensities exhibited similar patterns. After FMT, the increase in renin was markedly reduced, while the decrease in OPN was dramatically up-regulated, suggesting a renoprotective effect of FMT in rats.

## Discussion and conclusion

Oxalate is a simple organic acid that contributes to 80% of kidney stones. A deficiency of oxalate-degrading enzymes often causes foodborne oxalate accumulation in mammalian species. Only a small amount of oxalate is bioavailable for absorption because it exists in plants in the form of CaOx crystals. The majority of anionic oxalate is thought to be absorbed *via* a paracellular route in the gut (Hatch and Freel, 2005). Oxalate is eliminated by the kidney *via* filtration and is not secreted or reabsorbed beyond the proximal tubule. Thus, the oxalate concentration increases as water is reabsorbed along the nephron, reaching critical thresholds by the collecting duct, where it can crystallize along with calcium (Worcester et al., 2013). Fortunately, oxalate can be metabolized by gut microbiota such as *O. formigenes*, *Lactobacillus*, and *Bifidobacterium*. Specifically, oxalate can be microbially degraded to carbon dioxide and formate by *O. formigenes* through a simple two-step enzymatic reaction involving the enzymes formyl-CoA transferase and oxalyl-coenzyme A. Although probiotics, including *O. formigenes*, alone or in different combinations with facultative oxalate degraders, usually reduce urinary oxalate, they are not always effective. Furthermore, colonization by probiotic bacteria is commonly transient. In fact, the gut microbiota is composed of trillions of bacteria that reside primarily in the colon. In addition, microbial populations within the gut exist in functionally integrated, symbiotic networks, rather than as isolated functional species (Salazar and de Los Reyes-Gavilán, 2016). It is possible that the contribution of the gut microbiota to oxalate metabolism extends beyond oxalate-degrading bacteria alone. Few studies have attempted to explore the contribution of the whole gut microbiota to oxalate metabolism (Miller et al., 2016; Stern et al., 2019).

We transferred the whole gut microbiota from guinea pigs to SDRs. Before transplantation, we explored the reaction of guinea pig intestinal microbiota to an oxalate diet. As a result, a microbial network involving Muribaculaceae was identified in the gut of guinea pigs. Furthermore, we compared the species composition of microbiota in guinea pigs and SDRs. The results revealed that the relative abundance of Muribaculaceae in the intestine was much higher in guinea pigs than in SDRs. Bacteria in the family Muribaculaceae, belonging to the phylum Bacteroides, contain genes encoding the oxalate-degrading enzymes formyl-CoA transferase and oxalyl-CoA decarboxylase, which were first found in *O. formigenes*. These two genes represent a common pathway for oxalate degradation (Ormerod et al., 2016; Lagkouvardos et al., 2019). Oxalate is likely transported into the cell *via* a permease located adjacent to oxalyl-CoA decarboxylase. In addition, the number of OTUs shared between guinea pigs and SDRs indicates a high degree of transferability of bacteria.

FMT altered the gut microbiota of SDRs, as evidenced by increased microbial diversity in the SDR intestine. The increased similarity between the gut microbiota of SDRs and that of guinea pigs was also observed. In addition, whole gut microbiota transplantation activated the oxalate metabolism microbial network involving members of the Muribaculaceae *family* and other oxalate degraders in the gut of SDRs that received an oxalate diet. We also explored oxalate metabolism in the groups fed an oxalate-containing diet (groups OD + PBS and OD + FMT) and found that FMT resulted in a reduction in urinary oxalate excretion in SDRs. A lower CaOx crystal score in the kidney and a correspondingly lower CaOx crystal score in urine were observed in FMT recipients. Furthermore, we demonstrated that FMT is renoprotective in three respects. First, a reduction in urinary urea, urinary uric acid, urinary creatinine, serum BUN/creatinine ratio, and serum uric acid in group OD + FMT compared with group OD + PBS indicated that oxalate-induced kidney impairment was repaired to some extent. This was due to the degradation of oxalate in the intestine by oxalate degraders from a special microbial network; therefore, the toxicity of oxalate was prevented. Second, tubular dilatation and tubular atrophy induced by oxalate were alleviated in group OD + FMT. Third, a reduction in renin mRNA and up-regulation of OPN mRNA revealed that the kidneys of rats in group OD + FMT were better protected from oxalate toxicity and kidney stone-induced damage than the kidneys of rats in group OD + PBS. In summary, a microbial network involving Muribaculaceae and other oxalate degraders was activated by FMT in the intestine of SDRs, and oxalate was degraded by the microbial network in the gut. As a result, urinary oxalate excretion and CaOx crystals in urine and the kidney were reduced. In addition, oxalate-induced kidney damage was repaired to some extent.

Our work was another attempt to treat kidney stones with FMT based on previous research (Miller et al., 2016; Miller et al., 2017a; Stern et al., 2019). We pioneered the use of guinea pigs as donors of fecal transplants as they are herbivorous animals and their intestinal microbiota has been shown to have the ability to degrade oxalate (Argenzio et al., 1988). In addition, guinea pigs, as a common laboratory animal, are easily accessible. Miller et al. identified a microbial network that was related to oxalate metabolism in the intestine of *Neotoma albigula* (Miller et al., 2017b) and healthy human individuals (Miller et al., 2019). We discovered a similar microbial network that was represented by Muribaculaceae in the gut of guinea pigs. The family Muribaculaceae has been studied systematically in recent years, and it has been shown to have the ability to degrade oxalate (Lagkouvardos et al., 2019). Previous studies have focused only on the effects of FMT on oxalate metabolism. Our experiments revealed a reduction in CaOx crystal deposition in the kidney of SDRs. We also identified the renoprotective effects of the gut microbiota and found that FMT repaired oxalate-induced kidney damage to some degree. Our research could provide new ideas for the clinical treatment of kidney stones.

In conclusion, oxalate degradation ability could be transferred from guinea pigs to SDRs by FMT. The kidney was better protected as a result of the degradation of oxalate and reduction in CaOx crystal deposition.

## Data availability statement

The data presented in the study are deposited in the National Center for Biotechnology Information (https://www.ncbi.nlm.nih.gov/) SRA repository, accession number PRJNA933134.

## Ethics statement

The animal study was reviewed and approved by the experimental animal center of Wuhan University.

## Author contributions

YW and LG designed this study. JS, SX, YZ, and TW conducted animal experiments. YW, ZL, and CL collected, analyzed, and interpreted the data. YW and JS drafted the manuscript. TP, LG, and SX modified and revised the manuscript critically for intellectual content. All authors contributed to the article and approved the submitted version.
