# Supplementary material for: Increased abundance of bacteria of the family Muribaculaceae achieved by fecal microbiome transplantation correlates with the inhibition of kidney calcium oxalate stone deposition in experimental rats
Source: Front Cell Infect Microbiol. 2023 May 29;13:1145196. doi: 10.3389/fcimb.2023.1145196 (PMC10258309; doi:10.3389/fcimb.2023.1145196)
Supplement: Supplementary file 1 [file Table_1.docx]

**Additional file 1**

**Table S1: Primers used in this work**

| **Primers** | **Sequence (5ˈ→ 3ˈ)** |
| --- | --- |
| **GAPDH – F** | 5ˈTGCCAAGTATGATGACATCAAGAA3ˈ |
| **GAPDH – R** | 5ˈAGCCCAGGATGCCCTTTAGT3ˈ |
| **OPN – F** | 5ˈTGAGACTGGCAGTGGTTTGC3ˈ |
| **OPN – R** | 5ˈCCACTTTCACCGGGAGACA3ˈ |
| **Renin – F** | 5ˈACCAGGGCAACTTTCACTACGT3ˈ |
| **Renin – R** | 5ˈACCCCCTTCATGGTGATCTG3ˈ |
| **ACE – F** | 5ˈTTGTCTGTCACTGGAGCCTGAT3ˈ |
| **ACE – R** | 5ˈCACACCCAAAGCAATTCTTCGT3ˈ |

F, R: represent the forward and reverse primer respectively
